# Supplementary material for: A chromosome-scale genome assembly of the false clownfish, Amphiprion ocellaris
Source: G3 (Bethesda). 2022 Mar 30;12(5):jkac074. doi: 10.1093/g3journal/jkac074 (PMC9073690; doi:10.1093/g3journal/jkac074)
Supplement: jkac074_Supplemental_Figures [file jkac074_supplemental_figures.docx]

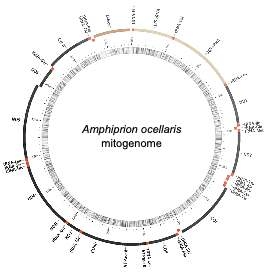


Figure S1. Mitochondrial genome annotation of *Amphiprion ocellaris*. The inner circle represents GC % per 5 bp of the mitogenome.

Figure S2. Phylogenetic trees constructed using (A) RAxML with 12,765 single copy genes from all 11 anemonefishes and their closest outgroup species *Acanthochromis polyacanthus*, (B) RAxML with 3,543 single copy genes from all 11 anemonefishes and six other fish species from across the teleost phylogenetic tree, (C) RAxML with 2,292 BUSCO genes from the same group of species used in A, (D) RAxML with 996 BUSCO genes from the same group of species used in B, (E) PhyloBayes with BUSCO genes from the same group of species used in C, and (F) PhyloBayes with BUSCO genes from the same group of species used in F. Numbers on each branching point are the bootstrap support (%) and the site concordance factor (%). These values were calculated only for non-outgroup species using the IQ-TREE algorithm.

Figure S3. Negative correlation between the divergence time and the number of synteny blocks between species pairs. The species pairing between *Amphiprion ocellaris* and *Amphiprion percula* is marked in green.

Figure S4. Dual synteny plot between all 24 chromosomes from *Amphiprion ocellaris* and *Amphiprion percula*. Chromosomal rearrangements such as translocations and inversions are shown as red ribbons, whereas blue ribbons represent unchanged regions.
